# Symptoms and risk factors of depression and PTSD in the prolonged COVID-19 pandemic: a longitudinal survey conducted from 2020 to 2022 in Japan

**DOI:** 10.1186/s12888-023-04670-7

**Published:** 2023-03-20

**Authors:** Yuko Fukase, Kanako Ichikura, Hirokuni Tagaya

**Affiliations:** Kitasato University School of Allied Health Sciences, Kitazato 1-15-1, Minami-ku, Sagamihara, Kanagawa 252-0373 Japan

**Keywords:** Mental health, COVID-19, Longitudinal studies, Depression, Stress disorders, Post-traumatic

## Abstract

**Background:**

The present study aimed to explore changes in depression and posttraumatic stress disorder (PTSD) among the general population during the prolonged COVID-19 pandemic and to investigate risk factors and adaptive/nonadaptive strategies.

**Methods:**

A web-based longitudinal survey was conducted across five timepoints from 2020 to 2022 in Japan. Depressive symptoms were measured using the Patient Health Questionnaire-9 (PHQ-9), PTSD was measured using Impact of Event Scale-Revised (IESR), and coping strategies were measured using Brief Coping Orientation to Problems Experienced (Brief COPE). Higher scores of PHQ-9 and IESR indicate more symptoms and Higher score of Brief COPE indicate that these means of coping are used very frequently.

**Results:**

A total of 1,366 participants (mean age = 52.76, SD = 15.57) were analyzed. Regarding levels of depression, PHQ-9 scores in 2022 were lower than in 2020 and 2021 (all p < 0.01). Regarding levels of PTSD, IESR scores in 2022 were lower than in 2021 among females (p < 0.001). Being younger (β = -0.08 and − 0.13, both p < 0.01) and engaging in self-blame (β = 0.12 and 0.18, both p < 0.01) increased PHQ-9 scores regardless of sex. For males, not working (β = 0.09, p = 0.004) and having suffered an economic impact (β = 0.07, p = 0.003) were risk factors for depressive symptoms, and active coping (β = -0.10, p = 0.005) was associated with decreased depressive symptoms. For females, substance use (β = 0.07, p = 0.032) and behavioral disengagement (β = 0.10, p = 0.006) increased depressive symptoms, and females did not show strategies that decreased the symptoms.

**Conclusions:**

Levels of depression might have increased in the early stages of the pandemic and decreased in January 2022. Although males need to improve their economic situation to decrease depressive symptoms, adaptive strategies might be difficult to identify due to the prolonged pandemic among both sexes. In addition, the pandemic might be a depressive event but not a traumatic event among the general population, at least in Japan.

## Background

The Coronavirus disease 2019 (COVID-19) has spread worldwide and the pandemic has led to social restrictions for the general population. During the early stages of the pandemic, meta-analyses showed levels of depression [1–7] and posttraumatic stress disorder (PTSD) [3, 8–12] increased among the general population. A previous study showed that age, social status, and economic impact of the pandemic were risk factors for depression and PTSD [1, 5, 13, 14]. On the other hand, some research that surveyed mental health between January and March 2020 reported that mental health sometimes improved during the stable period of the pandemic [15, 16]. Although much previous research investigated mental health in the early stage of infection, it is necessary to investigate levels of depression and PTSD and risk factors during the prolonged pandemic.

Moreover, previous research pointed out that mental health vulnerability varies by sex: females were found to have worse mental health than males before the pandemic [17] and to be more influenced by social restrictions due to the pandemic [18, 19]. Some coping strategies have been shown to be useful for maintaining mental health [20, 21]; however, the effect of coping strategies on mental health might differ by sex [22].

The present study aimed to explore the changes in depressive and PTSD symptoms from 2020 to 2022 in Japan. In addition, this study examined risk factors and adaptive/nonadaptive strategies associated with mental health by sex and provided ways of identifying individuals who need professional support in the context of the chronic stress associated with the pandemic. In Japan, strict lockdowns have never been implemented, but mild lockdowns have been implemented intermittently because the pandemic has exhibited a repeated pattern of expansion and contraction. Previous research conducted in 2020 and 2021 reported that the prevalences of depressive symptoms in Japan were 18.35% and 43.4%, respectively, and the rate might have increased before the pandemic [23–25]. Additionally, the rate of suicide in 2020 increased by 4.5% compared to that in 2019 [26]. Regarding sex, although the number of male suicides was 2 times as high as that of females in 2020 and 2021, the number of male suicides in 2020 and 2021 decreased from that in 2019; on the other hand, the number of female suicides in 2020 and 2021 increased from that in 2019 [26].

## Methods

### Study design and participants

We conducted a longitudinal survey that consisted of 5 web-based surveys administered by an online research company, Macromill, Inc., in Japan. Macromill, Inc. primarily conducts online market research for other companies. Macromill, Inc. and their partner companies have 10 million individuals registered as potential participants, from whom Macromill, Inc. can choose participants who meet the selection criteria for research. The 1st survey (T1) was conducted from 17 to 22 July 2020, the 2nd survey (T2) was conducted from 18 to 23 September 2020, the 3rd survey (T3) was conducted from 22 to 27 January 2021, the 4th survey (T4) was conducted from 17 to 21 September, and the 5th survey (T5) was conducted from 21 to 26 January 2022. The T1 survey was conducted on the earliest date that the Ethics Review Committee approved this study, and the deadline of the T1 survey was set to reach the target number of participants, as follows. The T2 to T5 surveys were conducted at regular intervals, and the deadline for each survey was set at 6 days.

From a pool of approximately 10 million individuals registered with Macromill, Inc. and their partner companies, we recruited participants who were between 20 and 79 years old and who lived in 13 out of the 47 prefectures in Japan. The 13 prefectures included those where COVID-19 had spread in April 2020, which was the first wave of the COVID-19 pandemic in Japan, and where the Government of Japan had implemented special measures. A quota sampling method was used to obtain age groups of equal size (i.e., groups of individuals in their 20s, 30s, 40s, 50s, 60s, and 70s), participants of both sexes (male and female), and participants with different employment statuses (full-time worker; no regular employment; and unemployed, including homemaker, retired, and jobless). All the participants received Macromill points for their participation; Macromill points are associated with the original point service provided by Macromill, Inc., and participants can trade these points for prizes or cash.

### Sample size and effect size

We planned to recruit 2,700 participants at T1 based on a calculation of the appropriate sample size [24]; however, the present study was a longitudinal study, and so it was difficult to control its sample sizes. We calculated effect sizes as follows: Cohen’s D (d) for the t test, Phi (φ) and Cramer’s V (V) for the chi-square test, and partial η2 (ηp2). With respect to d, 0.80 indicates a large effect size, 0.50 a medium effect size, and 0.20 a small effect size. Regarding φ and V, 0.50 represents a large effect size, 0.30 a medium effect size, and 0.10 a small effect size. Although no clear criteria are associated with ηp2, the higher the value of this measure is, the larger the effect size. For adjusted R2, 0.26 designates a large effect size, 0.13 a medium effect size, and 0.02 a small effect size.

### Measurements

The present study collected participants’ sociodemographic characteristics and included 3 questionnaires pertaining to depressive symptoms, PTSD symptoms, and coping strategies. PTSD symptoms were measured as part of the T2 survey, and all other sociodemographic characteristics and questionnaires were measured at all 5 time points.

The sociodemographic characteristics investigated included age, sex, the presence or absence of chronic illnesses, marital status, the presence or absence of children, employment status, household income (< 4 million JPY, 4–8 million JPY, and > 8 million JPY), and the economic impact for the COVID-19 pandemic. One million JPY was 9,400 USD in 2020 and, in Japan, the average annual household income was 5.52 million JPY, and the median income was 4.37 million JPY [27]. The economic impact of the COVID-19 pandemic was assessed by asking whether the participant perceived economic impacts of the pandemic.

Depressive symptoms were measured using the Japanese version of the Patient Health Questionnaire-9 (PHQ-9) [28, 29]. Participants were asked to indicate the frequency with which they had experienced depressive symptoms over the past 2 weeks. The PHQ-9 consists of 9 items scored on a four-point scale (0 to 3); the total score can range from 0 to 27, with higher scores indicating more depressive symptoms. The Japanese version of the PHQ-9 has been validated for depression in primary care, and the relationship with the mental component summary of the SF-8 was significant [29].

PTSD was measured using the Japanese version of the Impact of Event Scale-Revised (IESR) [30–32]. Participants were asked to indicate the frequency with which they had experienced PTSD symptoms over the past week. The IESR consists of 22 items scored on a five-point scale (0 to 4); the total score can range from 0 to 88, with higher scores indicating more PTSD symptoms. The Japanese version of the IESR has been validated for PTSD among nonsurvivors and survivors of traumatic events: high Cronbach’s alpha coefficients of the whole scale were shown, and participants with PTSD had significantly higher scores than those without PTSD [30].

Coping strategies were measured using the Japanese version of the Brief Coping Orientation to Problems Experienced (Brief COPE) [33, 34]. Participants were asked about the frequency with which they used various coping styles to deal with the social changes and inconveniences resulting from the COVID-19 pandemic at the time of completing the survey. The scale consists of 28 items and assesses 14 coping styles: self-distraction (e.g., “I turn to work or other activities to take my mind off things”); active coping (e.g., “I take action to try to make the situation better”); denial (e.g., “I refuse to believe that it has happened”); substance use (e.g., “I use alcohol or other drugs to make myself feel better”); use of emotional support (e.g., “I get emotional support from others”); use of instrumental support (e.g., “I get help and advice from other people”); behavioral disengagement (e.g., “I give up trying to deal with it”); venting (e.g., “I express my negative feelings”); positive reframing (e.g., “I try to find comfort in what is happening”); planning (e.g., “I think hard about what steps to take”); humor (e.g., “I make jokes about it”); acceptance (e.g., “I accept the reality of the fact that it happened”); and religion (e.g., “I try to find comfort in my religion or spirituality”). Each coping style is evaluated by reference to two items that are scored on a four-point scale (1 to 4); the total scores for each coping style can range from 2 to 8. Higher scores indicate that these means of coping are used very frequently. The Japanese version of the Brief COPE has been validated among Japanese workers: the relationships between coping styles and negative emotions, fatigue, concentration, and activity levels were significant [34].

### Statistical analysis

The present study analyzed participants who completed all 5 surveys. First, we compared the demographic characteristics of the analyzed participants and to those of participants who were excluded from the analysis. Sociodemographic characteristics at T1, mean PHQ-9 scores at T1 and mean IESR scores at T2 were compared using the chi-square test and two-sample t test. Subsequently, to compare the sociodemographic characteristics and coping strategies of the analyzed participants by sex, two-sample t tests and chi-square tests were conducted to explore their sociodemographic characteristics at T5 and mean Brief COPE scores at T5.

Thereafter, to examine changes in the level of depressive and PTSD symptoms across the timepoints of the survey, a mixed-model analysis of variance (ANOVA) was conducted with respect to the mean PHQ-9 and IESR scores including survey time as a within-subject factor and sex as a between-subjects factor.

Finally, to examine the influences of sociodemographic characteristics and coping strategies on depressive and PTSD symptoms, multiple linear regression analyses were conducted by sex using PHQ-9 and IESR scores at T5 as the dependent variables and sociodemographic characteristics and Brief COPE scores at T5 as the predictor variables.

The statistical significance level was set at p < 0.05, and effect sizes, Cohen’s D (d) for the t test, Phi (φ) and Cramer’s V (V) for the chi-square test, partial η2 (η_p_^2^) for ANOVAs, and adjusted R^2^ were used for multiple linear regression, as mentioned previously. All statistical analyses were conducted using IBM SPSS statistical software (Version 28).

## Results

### Comparison between participants and members of the drop-out group

**Fig. 1:**
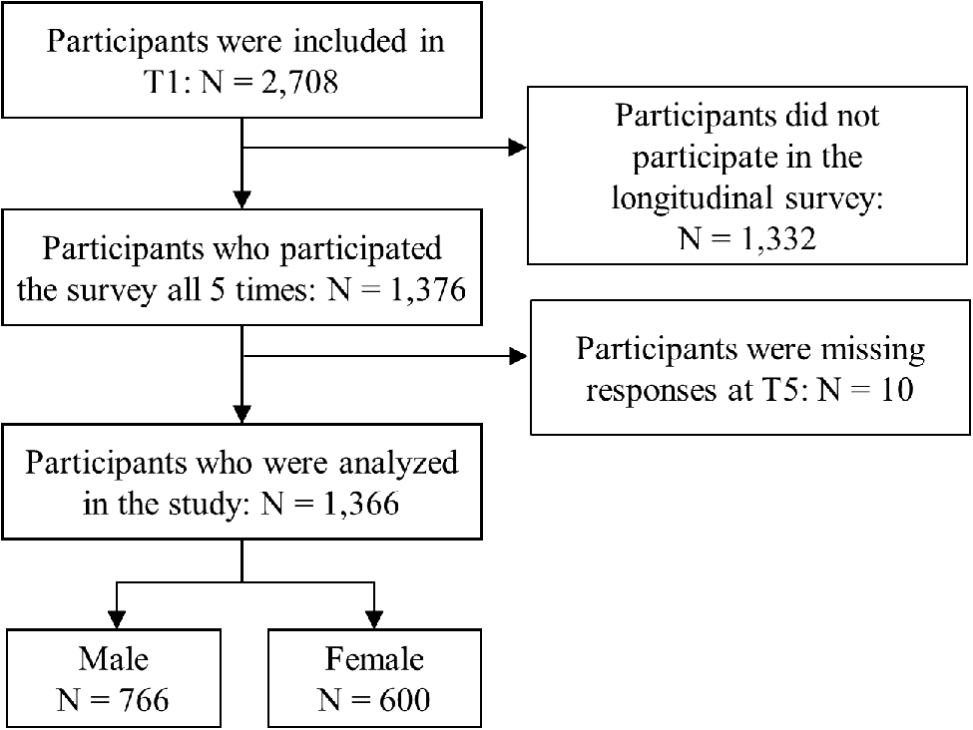
Flow chart of this study showing participant inclusion

A detailed description of study participant inclusion is shown in Fig. 1. A total of 2,708 participants were included in the 1st survey, of whom 1,342 were excluded from the analysis because they did not participate in the longitudinal survey (N = 1,332) or because they were missing responses (N = 10). Following these exclusions, a total of 1,366 participants were analyzed in the present study (for a valid response rate of 50.4%), of whom 776 were male and 600 were female.

A comparison between the final participants and the participants who were excluded from the analysis at T1 showed that the participants analyzed in the study were older (p < 0.001, d = 0.46) and included a large percentage of males (p < 0.001, φ = 0.12) and a large percentage of individuals with children (p = 0.007, φ = 0.05). Regarding employment status, a low percentage of participants were homemakers, and a large percentage did not work (p < 0.001, Cramer’s V = 0.09). Regarding household income, a large percentage of participants had a household income of more than 8 million JPY, and the participants included in the final analysis were less likely to provide no answer or an answer of “unknown” for household income (p < 0.001, Cramer’s V = 0.09). The participants included in the final analysis suffered a greater economic impact from the pandemic than participants who were excluded from the analysis (p = 0.001, d = 0.06). Regarding mental health, the analyzed group exhibited low PHQ-9 scores at T1 (p = 0.001, d = 0.13); however, there were no significant differences in PTSD symptoms at T2. The effect size of age difference was medium, those of employment status and household income were small, and other differences were very small.

### Participants’ sociodemographic characteristics and variables at T5 by sex

Sociodemographic characteristics and Brief COPE scores at T5 are shown according to sex in Table 1. The mean age among males was 53.73 ± 16.08 years, and that among females was 54.95 ± 14.89 years. Among the male, large percentages had chronic illnesses, were single, were not working, and had household income of less than 4 million JPY; on the other hand, low percentages did not have children and were homemakers. The effect size of employment status was medium, and those of chronic illnesses, marital status, children, and household income were small. Regarding Brief COPE, males used the strategies of self-distraction, active coping, emotional support, instrumental support, venting, positive reframing, planning, and acceptance less frequently and employed denial, substance use, and self-blame more frequently than females. The effect sizes of self-distraction, active coping, substance use, emotional support, instrumental support, and venting were small, and those of other coping strategies were very small.

**Table 1:** Sociodemographic characteristics and coping styles among participants at T5

	Male N = 766			Female N = 600			
	Mean/N	SD/%		Mean/N	SD/%	p	d/φ
Age	53.73	16.08		54.95	14.89	0.148	0.08
Chronic illnesses: With	131	17.1%		60	10.0%	< 0.0001	0.10
Marital status: Married	373	48.7%		401	66.8%	< 0.0001	0.18
Children: With	360	47.0%		387	64.5%	< 0.0001	0.17
Employment status							
Full-time worker	261	34.1%		198	33.0%	< 0.0001	0.48 ^a)^
Homemaker	7	0.9%		174	29.0%		
No regular employment	235	30.7%		190	31.7%		
Not working	263	34.3%		38	6.3%		
Household income							
< 4 million JPY	326	42.6%		184	30.7%	< 0.0001	0.12 ^a)^
4–8 million JPY	207	27.0%		199	33.2%		
> 8 million JPY	115	15.0%		99	16.5%		
No answer or unknown	118	15.4%		118	19.7%		
Economic impact: With	245	32.0%		194	32.3%	0.891	0.00
Brief COPE							
Self-distraction	4.13	1.45		4.47	1.51	< 0.0001	0.23
Active coping	4.79	1.54		5.04	1.43	0.002	0.17
Denial	3.22	1.39		3.08	1.25	0.046	0.11
Substance use	3.33	1.54		2.92	1.42	< 0.0001	0.28
Use of emotional support	3.62	1.46		3.97	1.53	< 0.0001	0.23
Use of instrumental support	3.57	1.44		3.90	1.51	< 0.0001	0.22
Behavioral disengagement	3.67	1.45		3.63	1.36	0.625	0.03
Venting	3.62	1.35		3.94	1.38	< 0.0001	0.23
Positive reframing	4.36	1.60		4.60	1.60	0.006	0.15
Planning	4.66	1.61		4.84	1.51	0.032	0.12
Humor	3.61	1.48		3.48	1.42	0.110	0.09
Acceptance	5.52	1.68		5.75	1.63	0.010	0.14
Religion	3.18	1.40		3.16	1.36	0.717	0.02
Self-blame	3.39	1.51		3.22	1.33	0.030	0.12

a) Cramer’s V

### Mean scores and prevalence of depressive and PTSD symptoms by sex

**Fig. 2:**
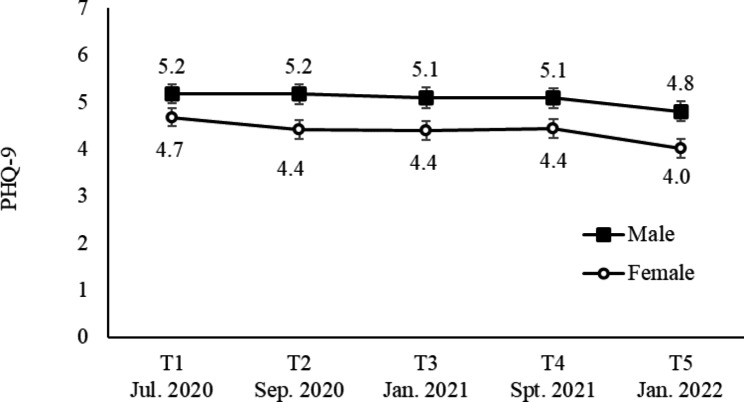
Mean PHQ-9 scores by sex from T1 to T5. Error bars show the standard error. The mean score of males was higher than that of females, and mean scores at T5 were lower than those at T1 to T4

Mean PHQ-9 scores by sex are shown in Fig. 2. The interaction between survey times and sex was nonsignificant (p = 0.577, η_p_^2^ = 0.001). The main effect of sex was significant (p = 0.012, η_p_^2^ = 0.005), and the mean score among males was higher than that among females. The main effect of survey time was significant (p < 0.001, η_p_^2^ = 0.006), and the mean score at T5 was lower than that at T1 (p < 0.001), T2 (p = 0.001), T3 (p = 0.003), and T4 (p < 0.001).

**Fig. 3:**
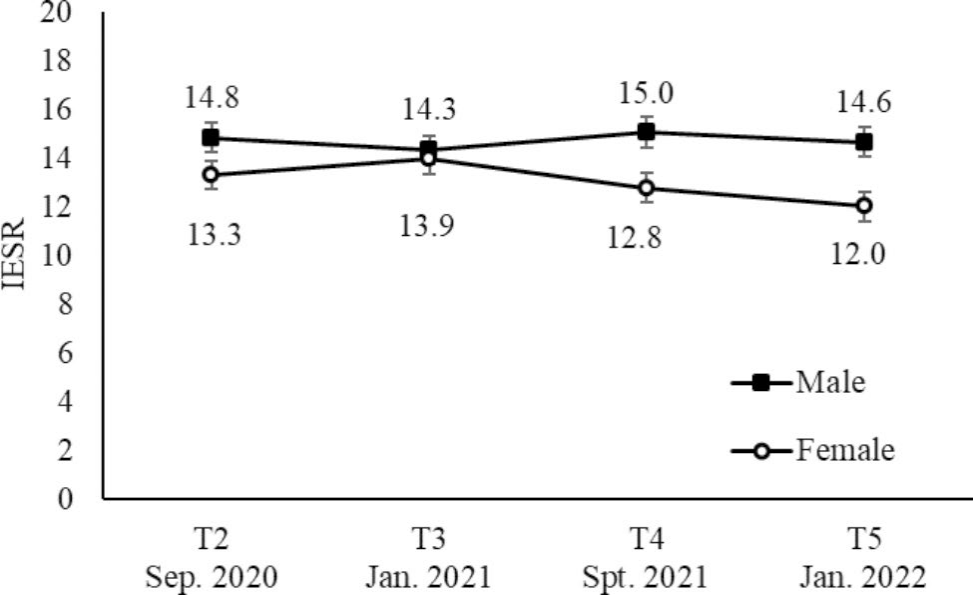
Mean IESR scores by sex from T2 to T5. Error bars show the standard error. The mean score of males was higher than that of females at T4 and T5. Among females, the mean score at T5 was lower than that at T3

Mean scores of PTSD symptoms by sex between T2 and T5 are shown in Fig. 3. The interaction between survey times and sex was significant (p = 0.003, η_p_^2^ = 0.003); males scored higher than females at T4 (p = 0.012) and T5 (p = 0.003), and the mean score at T5 among females was lower than that at T3 (p < 0.001). The main effects of sex were significant (p = 0.027, η_p_^2^ = 0.004), and males scored higher than females. The main effect of survey time was nonsignificant (p = 0.057, η_p_^2^ = 0.002).

### Risk factors and coping strategies for depressive and PTSD symptoms

The results of multiple linear regression analysis by sex concerning the prediction of depressive symptoms (PHQ-9) at T5 are shown in Table 2. Adjusted R^2^ scores were 0.64 in male and 0.56 in female and significant (p < 0.001 in both cases). Regarding risk factors for depressive symptoms, being younger was a risk factor for both males and females, and not working and having suffered an economic impact from the pandemic were risk factors for males. Regarding coping strategies, self-blame increased depressive symptoms for both males and females. Among males, active coping decreased symptoms, and venting increased symptoms. Among females, substance use and behavioral disengagement increased symptoms.

**Table 2:** Results of multiple linear regression analysis for predictors of PHQ-9 at T5

	Male			Female	
	β	p		β	p
PHQ-9 at T1	0.65	< 0.0001		0.59	< 0.0001
Age	-0.08	0.009		-0.13	< 0.0001
Chronic illness: With (reference: without)	0.02	0.389		0.03	0.226
Married (reference: single)	-0.06	0.095		0.00	0.989
Children: With (reference: without)	0.04	0.323		-0.01	0.820
Employment status (reference: full-time worker)					
Homemaker	0.01	0.799		-0.05	0.204
No regular employment	-0.01	0.813		-0.04	0.223
Not working	0.09	0.004		0.03	0.309
Household income (reference: 4–8 million JPY)					
< 4 million JPY	-0.02	0.550		0.04	0.302
> 8 million JPY	0.00	0.902		0.04	0.192
No answer or unknown	-0.02	0.409		-0.03	0.304
Economic impact: With (reference: without)	0.07	0.003		0.02	0.421
Brief COPE					
Self-distraction	0.04	0.163		0.02	0.574
Active coping	-0.10	0.005		0.01	0.757
Denial	0.00	0.925		-0.04	0.323
Substance use	-0.01	0.820		0.07	0.032
Use of emotional support	-0.06	0.110		-0.03	0.496
Use of instrumental support	0.00	0.904		-0.08	0.057
Behavioral disengagement	0.02	0.416		0.10	0.006
Venting	0.07	0.037		-0.03	0.424
Positive reframing	0.04	0.211		-0.02	0.716
Planning	-0.05	0.133		-0.05	0.295
Humor	-0.02	0.508		-0.07	0.057
Acceptance	0.04	0.200		0.04	0.314
Religion	0.07	0.017		0.03	0.400
Self-blame	0.12	0.001		0.18	< 0.0001

The results of multiple linear regression analysis by sex concerning the prediction of PTSD symptoms (IESR) at T5 are shown in Table 3. Adjusted R^2^ scores were 0.57 in male and 0.48 in female and significant (p < 0.001 in both cases). Economic impact was a risk factor for both males and females, and being younger was a risk factor for females. Regarding coping strategies, self-blame increased PTSD symptoms among males and females. Among males, self-distraction, behavioral disengagement, and venting increased symptoms, and the use of emotional support decreased symptoms. Among females, denial increased symptoms, and the use of instrumental support decreased symptoms.

**Table 3:** Results of multiple linear regression analysis for predictors of IESR at T5

	Male			Female	
	β	p		β	p
IESR at T2	0.53	< 0.0001		0.47	< 0.0001
Age	-0.04	0.286		-0.10	0.004
Chronic illness: With (reference: without)	0.02	0.326		0.01	0.750
Married (reference: single)	-0.01	0.871		-0.04	0.265
Children: With (reference: without)	-0.02	0.697		-0.01	0.729
Employment status (reference: full-time worker)					
Homemaker	0.01	0.741		0.05	0.187
No regular employment	-0.02	0.452		0.03	0.356
Not working	-0.01	0.775		-0.02	0.533
Household income (reference: 4–8 million JPY)					
< 4 million JPY	0.05	0.136		-0.04	0.275
> 8 million JPY	-0.03	0.239		0.07	0.057
No answer or unknown	-0.02	0.495		-0.05	0.136
Economic impact: With (reference: without)	0.06	0.011		0.09	0.005
Brief COPE					
Self-distraction	0.07	0.029		0.08	0.068
Active coping	-0.02	0.674		0.01	0.836
Denial	-0.02	0.654		0.08	0.044
Substance use	0.02	0.544		0.02	0.543
Use of emotional support	-0.09	0.047		0.01	0.832
Use of instrumental support	0.00	0.939		-0.12	0.015
Behavioral disengagement	0.07	0.024		0.06	0.126
Venting	0.08	0.032		0.02	0.609
Positive reframing	0.00	0.983		-0.04	0.331
Planning	-0.02	0.571		0.02	0.732
Humor	0.00	0.909		-0.06	0.091
Acceptance	-0.03	0.271		-0.06	0.170
Religion	0.04	0.191		0.06	0.105
Self-blame	0.21	< 0.0001		0.18	< 0.0001

## Discussion

The present study investigated depressive and PTSD symptoms, related factors, and coping strategies between 2020 and 2022. A total of 1,366 participants were analyzed, and the results showed that the level of depressive symptoms in 2022 was lower than that in 2020 and 2021. For both depressive and PTSD symptoms, males reported more symptoms than females. Risk factors and coping strategies related to depressive and PTSD symptoms are shown by sex.

### Characteristics of participants in the present study

Some sociodemographic characteristics of the participants in the present study were similar to those of the Japanese population in general [27, 35], such as the percentages of homemakers and jobless individuals females and household incomes among males and females. However, the percentage of males who were not working and that of single individuals among both males and females were larger than that the corresponding percentages among the Japanese population in general. Some studies have noted that being jobless and single might be risk factors for mental illness during the COVID-19 pandemic [36–38]. Thus, some degree of bias might have resulted from the quota sampling method. The excluded groups showed higher depressive levels than the included group. Consequently, it is possible that individuals with high levels of depressive symptoms might have been excluded from the analysis in the present study, and the participants in the present study might have included a large number of individuals who had some risk factors.

### Changes in levels of depressive and PTSD symptoms

Levels of depression might have increased due to the pandemic and subsequently decreased slightly in 2022. Because, the present study showed that levels of depressive symptoms in January 2022 were lower than in 2020 and 2021, and these scores in January 2022 were nearly the same as scores obtained before the pandemic [39, 40] rather than during the pandemic [23]. The same result was found for the number of suicides in Japan: the rate of suicides increased from July 2020 to June 2021, but the rate decreased from July 2021 to February 2022 [26]. Accordingly, the general population experienced depression during the pandemic, but it improved in 2022. However, it is possible that this decrease in depressive symptoms is temporary due to the increase in the number of suicides in March 2022 in Japan [26]; therefore, it is necessary to conduct a follow-up survey concerning mental health during and after the pandemic.

Regarding PTSD symptoms, although the symptoms of females decreased beginning in late 2021, IESR scores did not seem to decrease compared with the same scores before the pandemic [30]. Brunet, et al. [41] conducted a survey to investigate PTSD during the COVID-19 pandemic across countries, which indicated few participants met the clinical threshold for PTSD; accordingly, these authors noted that PTSD was not a common symptom associated with the COVID-19 pandemic. On the other hand, some studies concerning China, the Philippines, northern Iran, Poland, and Italy found high levels of PTSD symptoms during the pandemic [42–47]. Therefore, because PHQ scores increased in the early stages of the pandemic and decreased in the prolonged pandemic while IESR scores remained the same as before the pandemic, the pandemic might be a depressive event but not a traumatic event for the general population, at least in Japan, which has not conducted a strict lockdown but has conducted several mild lockdowns.

Additionally, the present study found that males exhibited higher levels of depression and PTSD than females; however, many studies have reported that being female is a risk factor for mental illness [17, 30, 48–51]. One potential reason for this difference is sampling bias, which is discussed in the limitations; thus, the conclusions of this study must be considered in combination with other studies.

### Sociodemographic characteristics and coping strategies for depressive and PTSD symptoms

Regarding depressive symptoms, the present study found that risk factors and nonadaptive strategies, that is, strategies that increased depressive symptoms, regardless of sex, were being young and engaging in self-blame, which is consistent with previous studies [1, 5, 13, 14, 24, 25]. Not working and perceiving an economic impact of the pandemic were risk factors among males; however, these economic factors were not related to symptoms among females. In addition, females employed only nonadaptive strategies; that is, these strategies decreased their symptoms, which is discussed below. On the other hand, males employed both nonadaptive strategies and adaptive strategies: males needed to take some action to improve their situation to decrease depressive symptoms. Additionally, although some previous studies have reported household income to be a risk factor [5], the present study did not find any relationship between household income and mental health; accordingly, people who were not working or who faced economic impacts might require mental health care regardless of their income.

Regarding PTSD symptoms, regardless of sex, risk factors and nonadaptive strategies included perceiving an economic impact of the pandemic and engaging in self-blame, respectively. To decrease PTSD symptoms, males need to receive emotional support from others, and females need help and advice from others.

However, the present study found that more nonadaptive strategies were used than adaptive strategies: blaming oneself for the pandemic was related to depression and PTSD regardless of sex, and becoming immersed, denying the situation, drinking alcohol and using drugs, behavioral disengagement, expressing negative feelings, and relying on religion as a distraction from negative feelings or a means of escaping the situation might lead to an increase in the symptoms of depression or PTSD.

Although some research conducted during the early stages of the pandemic showed that exercise and social support were effective in improving mental health [19, 52, 53], G Maggi, I Baldassarre, A Barbaro, ND Cavallo, M Cropano, R Nappo and G Santangelo [54] noted that it is difficult to discover adaptive strategies due to the prolonged nature of this stressful situation. Additionally, some of these nonadaptive strategies, that is denying the situation, drinking alcohol and using drugs, behavioral disengagement, and relying on religion, were identified as nonadaptive strategies before the pandemic [34]; accordingly, these strategies might be general nonadaptive strategies used to deal with daily stress, which is relevant since the pandemic has led to notable daily stressors for the general population.

### Limitation

The participants analyzed in the present study might have exhibited bias because the present study employed the quota sampling method, and a total of 50% of the participants were excluded from the analysis. Participants’ depressive and PTSD symptoms might not be reflective of the general population of Japan; in particular, young participants were excluded from the analysis more frequently, and previous studies have reported that being young is a risk factor related to mental health [20, 37, 52, 55–60].

As responses to the survey were self-reported, the mental health, behaviors and cognition of the participants were not observed. In particular, the measures of depressive and PTSD symptoms reported in the present study were not identical with diagnoses. In addition, it was difficult to examine certain sociodemographic factors, such as not working and not having regular employment, in detail in the present study.

Additional limitations of the study included the following: the study analyzed coping strategies cross-sectionally; the survey times could not be controlled; and the study used an online survey with incentives, which may have resulted in the inclusion of participants who felt compelled to participate to earn the economic incentive.

## Conclusion

The present study showed that levels of depression among the general population increased in the early stages of the pandemic and decreased in 2022. Regarding depressive symptoms, the risk factor among males was economic factors; efforts should be made to improve this situation to decrease depressive symptoms. However, especially for females, adaptive strategies might be more difficult to identify than they were during the early stages of the pandemic due to the prolonged pandemic and the diversity of perceptions of this stressful situation. Finally, PTSD symptoms were at the same level as before the pandemic, so the pandemic might be a depressive event but not a traumatic event among the general population, at least in Japan. However, because the potential remains for levels of especially depressive symptoms to increase at some point in the future, there is a need for long-term follow-up survey.

## Data Availability

The datasets used during the current study are available from the corresponding author on reasonable request.

## List of abbreviations

COVID-19: Coronavirus disease 2019
PHQ-9: Patient Health Questionnaire-9
IESR: Impact of Event Scale-Revised
Brief COPE: Brief Coping Orientation to Problems Experienced
ANOVA: Analysis of variance
